# Partial Paralysis and Want of Co-ordination from Irritation of the Genital Organs

**Published:** 1880-05-20

**Authors:** Lewis A. Sayre

**Affiliations:** New York


### *PARTIAL PARALYSIS AND WANT OF CO-ORDINATION FROM IRRITATION OF THE GENITAL ORGANS.*

BY LEWIS A. SAYRE, M.D., OF NEW YORK.

This subject of irritation of the genital organs, and the very serious results that are apt to occur in connection with it, is not, I am sorry to say, appreciated by the profession at large to the extent that it should be; though I for one, at least, have done all that I could to direct attention to the matter. It is one of such great importance that you cannot be too constantly on your guard respecting it; the truth of which statement, I think, is well illustrated by the remarkable case which I will now read to you. "John English, aged forty-six, native of England, widower, clerk by occupation. Admitted to Workhouse Hospital, Blackwell's Island, December 23, 1878. Patient had been at work for a week, as a prisoner. On the 23d he was noticed to be restless and uneasy, and finally in the evening he fell from his bunk in a fit. During the next forty-eight hours he had several convulsions (eight or nine altogether), and in the intervals between them lay in a semi-comatose condition, showing no consciousness except to stir a limb when pinched. Pulse 120, temperature 101.5° F., respiration 18. Swallowed nothing, and passed fæces in bed. He continued in this state until the evening of December 25th (the temperature in the meanwhile having fallen to 100° F.), when a string was discovered passed twice around the penis just behind the corona and tied, the long prepuce serving to conceal it from notice. While this was not sufficiently tight to cause occlusion of the urethral canal, it had given rise to the formation of a firm, indurated band, which did not altogether disappear for four or five days after the string had been removed. Within one hour from the removal of the latter the man sat up and asked for milk, and from this time remained perfectly well, being under observation for fully three months. He declared that he remembered nothing that had taken place during the three days previous. This patient had never before been subject to 'fits,' and although he confessed that he was moderately addicted to drink denied venerial, and said that he had led a 'virtuous life' since the death of his wife, two years before."

In children a certain amount of agglutination between the glans penis and the prepuce covering it is normal in early life, while the prepuce gradually unfolds until the age of puberty, when the glans can be uncovered without difficulty. But sometimes, instead of merely an agglutination, there is actual adhesion, and, as a result of this, there accumulates behind the corona a ring of hardened smegma, which consists principally of earth deposits from the urine. This concretion, which is almost as hard as stone, being tightly imprisoned by the narrow prepuce, produces so much irritation that the penis is kept in a constant state of erection, and the entire nervous system of the child becomes profoundly affected. On looking at the little fellow now before you, you observe the marked pallor of his countenance, and this is a characteristic feature of all these cases, on account of the constant worriment and strain to which the patient is subjected. When we come to examine the penis we do not wonder that he has a continual desire to pass his water, for even in this state of quiescence we find that the meatus and the surrounding mucous membrane are very red, as though there were urethritis present. When I touch with my finger the orifice of the urethra, you notice that an instant spasm of both lower extremities is produced by the irritation, and hence you can readily see how the constant pressure of the child's clothing will act in keeping up an excitation and a more or less permanent state of erection in the penis. You all know what a powerful impression an ordinary erection, lasting but a few moments, makes upon the whole organism in the adult, and from this you can judge what an enormous strain upon the system must be constantly kept up in the case of a young child where there is such a state of affairs as exists here.

There is but one way to effect a cure in such a case as this, and that is by operating upon the organ where the seat of difficulty lies. In some instances it is sufficient merely to slit up the narrow prepuce, and thus release the glans from its imprisonment; but where there is great redundancy of this part, as in the present patient, it is necessary to remove a considerable portion of it by circumcision. In performing the operation, I first draw the prepuce well forward with the fingers, and grasp it in front of the glans with a pair of flat-bladed forceps made for the purpose, when I snip off cleanly with the scissors all the portion in front of the forceps. I can now readily draw back the integument over the glans, but not the mucous membrane, which has not been reached in the cutting, and so still remains tightly around the part. Should we go no further in our operation than this, we should do our patient more harm than good; and, accordingly, having inserted a grooved director under the mucous membrane, I divide it along the dorsum of the penis for a sufficient distance, and then pull it back from the glans, at the same time breaking up such adhesions as are met with. I have now uncovered the glans, and you observe the ring of hardened smegma which is always found behind the corona in these cases, and, having cleared that all away, I take the glans between the fingers and tear the frænum in two. The head of the penis is therefore at length completely barred. It is sometimes necessary to take a stitch or two between the edges of the skin and mucous membrane, but it is always better to avoid this if possible. By cutting the mucous membrane back to the corona, and thus dividing or tearing the frænum, as you have just seen me do, we are usually able to bring the two edges into close opposition, and sutures are thus rendered unnecessary.

The dressing to be applied after the operation is a simple one. Drawing back the prepuce sufficiently to expose the whole glans, and bringing the edges of the skin and mucous membrane close in contact, I apply a little styptic cotton or persulphate of iron to the torn frænum, and place small strips of old linen rag behind the corona in such a way that the cut edges of the skin and mucous membrane will be retained in their proper position. These should not be tied, but simply laid on loosely, so that if there is any swelling of the parts following the operation the penis will not be tightly constricted and its circulation interfered with. They should then be allowed to remain in position, the blood drying in them, until they fall off of themselves, when, if all goes well, it will be found that the parts have all perfectly healed, and the operation has been a complete success. I next place, externally, a little more styptic cotton, so as to assist in coagulating the blood without interfering with primary adhesion between the cut edges, which is sometimes the case if it is applied directly to the raw surface. In order to protect the exposed glans from friction by the clothing or other pressure, we put a handkerchief or piece of cotton batting, twisted somewhat into the shape of a ring, around the penis, and lay a linen cloth wet with cold water (which can be renewed as often as is desired without disturbing the rest of the dressing) just over the glans. Finally, an ordinary diaper is pinned on, in order to keep the whole in position.

If the mother will be kind enough to bring this child back at the end of a fortnight, I am sure that even in that short time we shall find a marked improvement in the case. In the meanwhile I can, from a very large experience with similar cases, prophesy with confidence that the little patient will sleep quietly from the very first night, and be no more trouble with any nightmares; and if it should turn out that what we have now done for him should be the means of restoring function to his paralyzed muscles, it will certainly be a very extraordinary way of curing "chronic disease of the knee-joint."

In connection with this case I will refer briefly to another, of similar character as regards the origin of the trouble present, though somewhat different in its manifestations, which, as many of you will no doubt recall, was at the clinic a few weeks ago, and which I mention now because it presented some points of more than ordinary interest. The patient, you remember, was the child of a physician, residing at a distance, and was brought here by the father himself. This was the second occasion on which the latter had consulted me in reference to him; the first time having been more than six months before, in April last. At that time he was four years of age, and had never been able to walk. He could not even stand without assistance, and then his legs were always more or less flexed, as it was impossible for him to straighten them out when any weight was resting upon them. He was large and fat, but his flesh was flabby, and he presented the same pale and waxy appearance that the child just operated upon does, while he lacked to a very marked degree the power of controlling his muscles. This was especially the case as regards those of the tongue; so that when he attempted to talk he always stammered badly. Nevertheless he was quite as intelligent and quick-witted as any child of his age, and, as evinced by the expression of his countenance, comprehended perfectly everything that was said to him, though he was, unfortunately, unable to reply. He never rested well when asleep, and frequently seemed to suffer from nightmares. Moreover, it was ascertained that he had almost constant priapism, and that there was a continual dribbling of urine from the penis, while he was unable to eject a full stream in the right direction.

On making an examination of the genitals, I found that it was impossible to uncover the glans, as the prepuce was narrow and firmly adherent, just as in the case of to-day. From previous experience, therefore, I felt justified in concluding at once that here was the source of all the reflex irritation present, and advised in the strongest terms that the doctor should have him circumcised. Accordingly, he was taken home, and this was done, as was thought, but, unfortunately, it was performed in such an imperfect manner by the surgeon who attempted it that it resulted in no permanent benefit; for the prepuce was merely slit up along the dorsum of the penis, while the mucous membrane remained intact, and the glans was really never uncovered. Yet immediately after the operation, incomplete as it was, the stammering disappeared, and the child for the first time in its life was able to speak distinctly. The parents were delighted, and on account of the improvement which resulted so quickly they felt assured that their darling would soon be restored to perfect health. But, alas, their hopes were short-lived, and at the end of three months the child was as bad in every respect as he was before. It was on account of this relapse that they came to consult me the second time.

When I examined the penis I found that the prepuce was firmly adherent, as before, and that there was a long projecting piece of integument, which had been left by the operation. You remember that I then in your presence performed circumcision in the proper manner, just as I have done to-day, and as it ought to have been done at first. When the glans was uncovered, the father remarked that it was nearly twice as large as it had been at the time of the first operation, a few months ago; which shows that its regular development had been interfered with by the abnormally narrow and constricted prepuce. Four hours after the operation I called to see the little patient at his hotel, just as the family were on the point of leaving town, and when I entered the room he immediately called out to me, "How do you do, Dr. Sayre?" in the most unhesitating and distinct manner, and you will recollect that just before the operation he could hardly articulate distinctly. Finally, I wish to bring back to your minds to-day still another case, which was here just before the physician's child, and which was of a more aggravated character than either it or the one operated on this morning. This was a boy from Norfolk, Virginia, fourteen years of age, who had been brought to me first nearly two years previously, by Dr. Norcom, of North Carolina. Up to that time he had been supposed to be hopelessly idiotic; while he had never been able to feed himself, to speak an intelligible word, to walk, or even to stand up, so that he required the constant services of a nurse, both by day and by night. As I found, however, that he was subject to continual priapism, and that the prepuce was constricted and adherent, I believed there was still hope for him, and, accordingly, performed circumcision. Directions having been given for the frequent manipulation of the feeble muscles, and the application of electricity to them, in addition, he was sent home, with the request that he should be brought back to New York about once in six months, so that I could observe the progress in the case. From the first there has been a gradual but altogether satisfactory improvement, and for some time now he has been able to get about very nicely with the assistance of a wheel-crutch; while his faculties seem to have become developed, and he is able to speak with considerable facility. These cases, I think, will be sufficient to convince you of the very great importance of this matter; and if the consideration of them will enable any of you to discover the real cause of the phenomena presented by patients suffering in this way, and to adopt the proper method of treating them, I will not have talked in vain upon the subject.—*Boston Med. Surg. Journal.*

